# SGLT2 inhibitors in autoimmune diseases: emerging therapeutic potential and clinical challenges

**DOI:** 10.3389/fimmu.2025.1589341

**Published:** 2025-07-03

**Authors:** Taimin Luo, Liaoyun Zhang, Kun Tu, Gen Li, Hao Su, Guanli Gong, Yilan Huang, Min Li, Xuping Yang

**Affiliations:** ^1^ Department of Pharmacy, Chengdu Seventh People’s Hospital (Affiliated Cancer Hospital of Chengdu Medical College), Chengdu, China; ^2^ Department of pharmacy, Sichuan Provincial Woman’s and Children’s Hospital & The Affliated Women’s and Children’s Hospital of Chengdu Medical College, Chengdu, Sichuan, China; ^3^ Department of Pharmacy, The Affiliated Hospital, Southwest Medical University, Luzhou, China; ^4^ School of Pharmacy, Southwest Medical University, Luzhou, China

**Keywords:** SGLT2 inhibitors, autoimmune diseases, T cells, metabolic reprogramming, inflammatory response

## Abstract

Autoimmune diseases (AIDs) are conditions where the immune system mistakenly attacks self-antigens, leading to tissue and organ damage. The exact mechanisms underlying AIDs pathogenesis remain unclear, and effective treatments are currently limited, posing significant therapeutic challenges. Recent studies suggest that targeting T cell immune metabolism could be a promising approach for treating AIDs. Repurposed type 2 diabetes mellitus (T2DM) medications, which modulate immune metabolic processes, have shown potential in various inflammatory conditions. Sodium-glucose cotransporter-2 (SGLT2) inhibitors, a novel class of oral antidiabetic agents, not only regulate metabolic dysfunction but also offer protective effects on the heart and kidneys. Emerging preclinical evidence indicates that SGLT2 inhibitors possess immunomodulatory properties, highlighting their potential in enhancing T cell-mediated autoimmune therapy. Clinical studies further validate that SGLT2 inhibitors significantly reduce the risk of chronic kidney disease (CKD) progression in non-diabetic patient groups, such as those with chronic glomerulonephritis like IgA nephropathy. This review aims to evaluate current preclinical and clinical research on the impact of SGLT2 inhibitors on the immune system and explore their mechanisms of action relevant to treating AIDs.

## 1 Introduction

Autoimmune diseases (AIDs) occur when the immune system mistakenly attacks the body’s own tissues and organs, leading to conditions such as rheumatoid arthritis (RA), systemic lupus erythematosus (SLE), multiple sclerosis (MS), and vasculitis (1, 2). Epidemiological data indicate that the prevalence of AIDs has been steadily increasing over the past few decades, affecting approximately 5% to 9% of the global population (3). While the exact mechanisms of AIDs remain unclear, they are believed to involve a combination of immune system dysfunction, genetic predisposition, and environmental factors. Conventional treatments for AIDs include analgesics, non-steroidal anti-inflammatory drugs (NSAIDs), and corticosteroids (2). In recent years, immunosuppressants and biologics have shown significant therapeutic potential in clinical practice (4). However, achieving a complete cure remains challenging, and the broad, non-specific effects of these treatments often lead to toxic side effects, imposing a substantial burden on patients. Therefore, there is an urgent need to explore new and potentially effective therapeutic options.

## 2 Targeting immunometabolism

**Figure 1:**
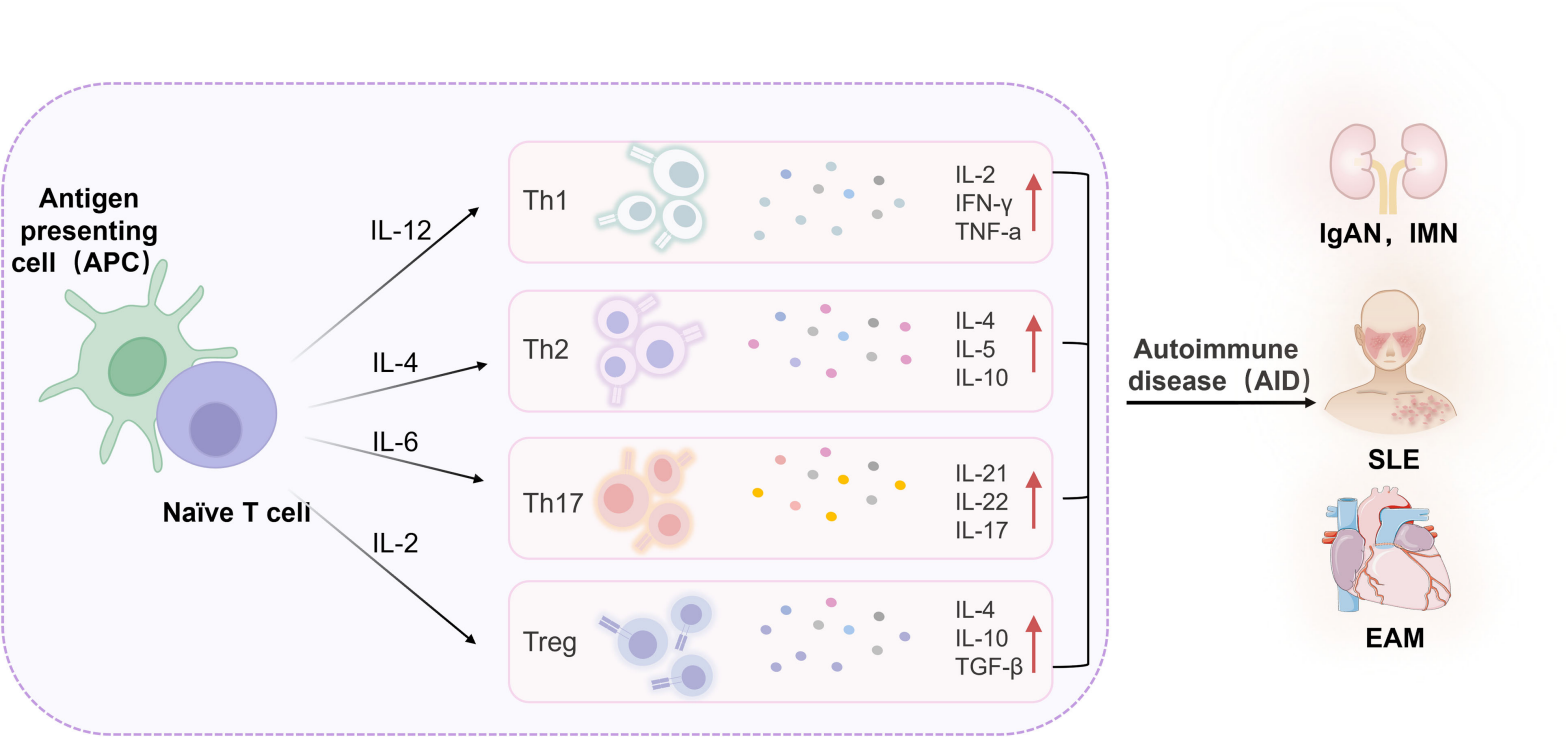
Inflammatory cytokines in AIDs. IL, Interleukin; Th, T helper cell; Treg, Regulatory T cell; IFN-γ, Interferon-gamma; TNF-α, Tumor necrosis factor-alpha; TGF-β, Transforming growth factor-beta; IgAN, Immunoglobulin A nephropathy; IMN, Idiopathic membranous nephropathy; EAM, Experimental autoimmune myocarditis; SLE, Systemic lupus erythematosus.

Immune system dysfunction is a primary driver of AIDs, involving the activation of self-reactive T cells and B cells, the downregulation of regulatory T cells (Tregs), and the release of pro-inflammatory cytokines, collectively disrupting the body’s autoimmune tolerance (5–7). Consequently, the immune system mounts an excessive response against self-antigens, precipitating AIDs. Upon antigen stimulation, naive CD4^+^ T cells undergo activation, proliferation, and differentiation into various subsets, including Th1, Th2, Th17, and Tregs (8, 9). Th1 cells predominantly secrete cytokines such as interferon-γ (IFN-γ), tumor necrosis factor-β (TNF-β), and interleukin-2 (IL-2), when overactivated, which contribute to organ-specific AIDs. Th2 cells produce cytokines like interleukin-4 (IL-4) and interleukin-10 (IL-10), which have anti-inflammatory properties but can lead to systemic AIDs when overactivated (10). Th17 cells, a key pathogenic subset, show increased infiltration at disease lesions, correlating positively with disease severity (11). In the early stages of AIDs, Th17 cells promote inflammation by producing pro-inflammatory cytokines. Tregs exert inhibitory effects on T cell proliferation, activation, and cytokine secretion, maintaining autoimmune tolerance by limiting B cell accumulation. Normally, a balanced state between Th17 and Treg cells exists; however, dysregulation of this balance can precipitate disease onset (**Figure 1**).

**Figure 2:**
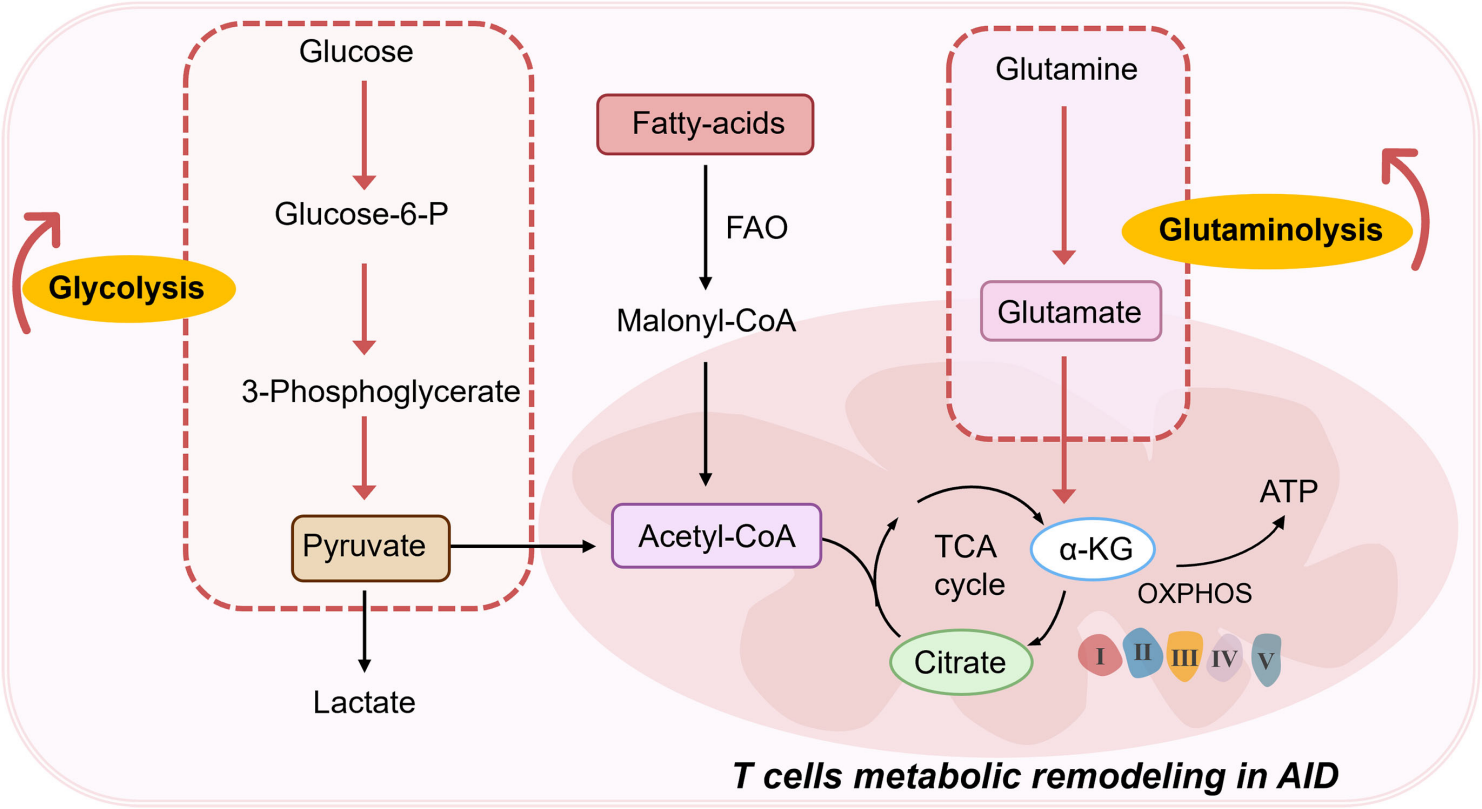
T cells metabolic remodeling in AIDs. FAO, Fatty acid oxidation; TCA, Tricarboxylic acid; α-KG, α-Ketoglutaric acid; OXPHOS, Oxidative phosphorylation; ATP, Adenosine triphosphate.

Normally, Th17/Treg cells maintain a relative balance, but when this balance is disrupted, disease can ensue. One characteristic of AIDs is abnormal T cell activation, and T cell function is closely related to metabolism. Naive T cells primarily rely on oxidative phosphorylation (OXPHOS) and fatty acid oxidation (FAO) for energy. Upon activation, T cells switch to glycolysis to rapidly meet energy demands, a process known as metabolic reprogramming (12–14) (**Figure 2**). This shift supports T cell proliferation and effector functions. Metabolic reprogramming not only affects T cell activation and proliferation but also determines the differentiation direction of T cell subsets. Upon antigen activation, T cells undergo metabolic reprogramming, shifting their energy metabolism from OXPHOS to faster energy-producing glycolysis and glutaminolysis to quickly meet the energy demands of cell activation, differentiating into effector T cells (Teff). On the other hand, memory T cells and Tregs rely on OXPHOS and FAO to maintain their survival and differentiation. The imbalance between helper T cell subsets is closely related to SLE disease activity and Systemic sclerosis(SSc) (15, 16). The increase in the Th17/Treg ratio and the expansion of Th1/Tfh cells are particularly important in the pathogenesis of SLE (17, 18). Glycolysis and mitochondrial OXPHOS levels in SLE CD4^+^ T cells are significantly enhanced (19). These metabolic reprogramming processes lead SLE CD4+ T cells to differentiate into Th1, Th17, and Tfh cells subsets (20). Additionally, acetyl-CoA produced in the tricarboxylic acid cycle (TCA cycle) can promote the production of inflammatory T cells by upregulating histone acetylation, mediating the onset and progression of SLE (21). Additionally, in RA, mitochondrial OXPHOS in T cells is impaired, causing glucose metabolism to shift from glycolysis to the pentose phosphate pathway, resulting in decreased ATP levels in RA T cells and triggering fatty acid synthesis in T cells. On the other hand, acetyl-CoA derived from citrate can promote tubulin acetylation (22). These shifts in metabolic pathways and increased epigenetic modifications cause RA T cells to exhibit high tissue invasiveness and pro-inflammatory effects. Synovial membranes in RA patients demonstrate pronounced infiltration of aberrantly activated CD4^+^ T lymphocytes that drive robust secretion of inflammatory mediators (23).Comparative analysis reveals distinct metabolic reprogramming in RA T-cell subsets: CD8^+^ T lymphocytes from RA patients demonstrate significantly upregulated glycolysis- and fatty acid synthesis-related gene expression alongside suppressed oxidative phosphorylation pathways compared to healthy donor counterparts, with concurrent elevation of lactate dehydrogenase levels (24). Parallel investigations in RA murine models (25) identify CD4^+^ T cells display a hypermetabolic state characterized by heightened activation of mammalian target of rapamycin complex 2(mTORC2); Notably, early-phase glycolytic inhibition substantially attenuated synovial inflammation, underscoring the therapeutic potential of metabolic modulation (25). **A** study aimed to understand RA-specific signatures in CD4^+^T cells using multi-omics data revealed that the methylomic changes, driven by RA heritability-explaining variants, shape the differential expression of a substantial fraction of differentially expressed genes in CD4^+^ T cells in patients with RA (26). Furthermore, epigenetic regulation through DNA methylation dynamically governs T-lymphocyte differentiation and trafficking, while activated T cells reciprocally amplify inflammatory cascades via IL-2 overproduction, creating a pathogenic feedback loop that perpetuates RA progression (27).

Several preclinical studies have demonstrated the therapeutic advantages of targeting T cell metabolism in autoimmunity. Approaches include using allosteric activators of pyruvate kinase (28), inhibiting OXPHOS with oligomycin (29), and targeting glycolysis and OXPHOS using inhibitors such as 2-deoxy-D-glucose (30) and glutaminase (31). Normalizing CD4^+^ T cell metabolism has been shown to reduce T cell activation, proliferation, and cytokine production. Metformin, a drug used in T2DM that targets cellular metabolism, has exhibited promising therapeutic potential in AIDs such as RA, SLE, and MS (32–36). Recent clinical trials indicate that metformin can reduce the onset risk in SLE patients (34). Additionally, the thiazolidinedione inhibitor pioglitazone has demonstrated immunomodulatory and anti-inflammatory effects in MS patients (37, 38). These findings underscore the potential of targeting metabolic changes in pathogenic T cells, allowing for the repurposing of T2DM drugs for autoimmune therapy.

## 3 Effects of sodium-glucose cotransporter 2 inhibitor in AIDs

SGLT2 inhibitors are a novel class of antihyperglycemic agents that improve glycemic control in diabetic patients by reducing renal glucose reabsorption, lowering the renal glucose threshold, and increasing urinary glucose excretion (26, 39). Beyond their glucose-lowering effects, SGLT2 inhibitors have garnered attention for their off-target benefits, particularly their cardioprotective and renoprotective effects (40, 41). Recent research highlights their potential to modulate immune responses, particularly in T cell-mediated conditions. (42–45). Moreover, the renoprotective benefits of SGLT2 inhibitors have been extended to patients with non-diabetic CKD (e.g., IgA nephropathy) (46). In recent years, preclinical studies have shown that SGLT2 inhibitors have immunomodulatory effects and can inhibit the effector functions of human T cells. In addition, several small-scale exploratory clinical studies have recently found that SGLT2 inhibitors therapy may be beneficial for patients with AIDs. Therefore, with their immunomodulatory and cardiorenal protective properties, SGLT2 inhibitors have become an attractive candidate for the treatment of patients with AIDs. The following sections explore the effects of SGLT2 inhibitors in specific autoimmune conditions.

### 3.1 SGLT2 inhibitors and SLE

SLE is a diffuse connective tissue disease characterized by autoimmune inflammation affecting multiple organ systems (47–49). Key clinical features of SLE include the presence of various autoantibodies, such as anti-nuclear antibodies, and widespread systemic involvement (50). Lupus nephritis (LN) is one of the most common and severe complications of SLE, marked by renal inflammation that can progress to end-stage renal disease (ESRD). Additionally, the elevated risk of cardiovascular events significantly contributes to the mortality of long-term SLE patients (51, 52). Current treatment strategies primarily involve glucocorticoids and other immunosuppressants as first-line therapies, with renin-angiotensin-aldosterone system inhibitors (RAASi) providing additional renal protection for LN patients. However, despite these treatments, some LN patients remain at risk of developing ESRD (53). Therefore, developing novel therapeutic agents that offer renal protection, reduce proteinuria, and attenuate progressive renal failure in LN patients is of substantial importance.

A small clinical trial by Morales (54) evaluated the renal therapeutic potential of SGLT2 inhibitors in LN patients (**Table 1**), given their notable cardiorenal protective effects. The trial involved five LN patients who were already receiving immunosuppressive therapy and were additionally administered Empagliflozin at a dose of 10 mg/day. The baseline proteinuria averaged 2.2 g/day, and the study monitored changes in glomerular filtration rate (GFR), proteinuria, and serum albumin over an 8-week period. The results indicated a significant 49.9% reduction in proteinuria within the initial 8 weeks of treatment, while GFR remained relatively stable. These preliminary findings suggest that SGLT2 inhibitors, in conjunction with standard care involving RAASi, may offer substantial potential in reducing proteinuria and providing renal protection in LN patients. The combination of SGLT2 inhibitors with RAASi could present an effective strategy for managing residual proteinuria in this patient population.

**Table 1:** Clinical trials and animal studies on SGLT2 inhibitors in AIDs.

AID	Study object	SGLT2I dose	Duration	Main Results	Ref
Human study					
SLE	Patients with SLE with/without LN	Dapagliflozin 10mg/kg	6 months	**Primary outcomes** Any adverse events (AEs) **Secondary outcomes** Change in SLEDAI score at last visit Change in 24-hour UPRO at last visit Change in haemoglobin at last visit Change in eGFR at last visit 6-month eGFR slope	(55)
	LN patients	Empagliflozin 10 mg/day	8 weeks	**Primary outcomes** Proteinuria decrease Glomerular filtration rate	(54)
	LN Patients With Chronic Kidney Disease	Dapagliflozin 10mg/day	24 months	**Primary composite endpoint** eGFR reduction **Secondary outcome measures** eGFR, UPC, ESKD, Fasting glucose, Hba1c, Lipids, Anti-dsDNA, C3, Memory B cells, MiR-148a, BACH1, BACH2, PAX5,Clinical relapses, Urinary tract infection, Kketoacidosis, Genital infection, Acute kidney injury	NCT06155604 (ongoling)
	LN patients	Dapagliflozin 10mg/day	6 months	**Primary composite endpoint** Serum urea level, Serum creatinine level, Serum uric acid level	NCT06113900 (ongoling)
	LN patient with or without diabetes	Dapagliflozin 10 mg/day	1 year	**Primary composite endpoint** eGFR, Coronary calcification, Erythropoietin level, Hepcidin level; **Secondary outcome measures** ECCHO parameters, Body weight	NCT05748925 (ongoling)
	LN Patient with Bone and Mineral Disease	Dapagliflozin 10 mg/day	1 year	**Primary composite endpoint** eGFR, S.creatinine, Osteoporosis, Erum calcium and phosphorus, Bone turnover markers; **Secondary outcome measures** Blood pressure Body weight	NCT05704088 (ongoling)
IgAN	patients with IgAN	Dapagliflozin 5 or 10 mg/day	2.1 years	**Primary composite endpoint** Dapagliflozin group compared to placebo group (HR 0.29; 95% CI 0.12–0.73; P=0.005) **Secondary kidney-specific outcomes** Dapagliflozin group compared to placebo group (HR 0.24; 95% CI 0.09–0.65; P=0.002) **Progression to ESKD** Dapagliflozin group compared to placebo group (HR 0.30; 95% CI 0.11–0.83; P=0.014).	DAPA-CKD, NCT03036150
Animal study					
LN	MRL/lpr mice	Empagliflozin 10mg/kg	10 week	**Empagliflozin vs. control** Anti-dsDNA IgG(P<0.001) Total IgG(P<0.01) Serum creatinine (P<0.001) Urinary protein excretion( P<0.001) Renal histological alterations	(56)
IMN	rat model of membranous nephropathy (MN)	Canagliflozin-treated 10 mg/kg/day	8 weeks	**Cangliflozina-treated vs. control** Blood urea nitrogen Serum creatinine SUA: senum uric acid Glomerular pathological damage Renal immune complex deposition Podocyte injury	(57)
Autoimmune-mediated cardiac injury	Experimental Autoimmune Myocarditis	Canagliflozin-treated 30 mg/kg/day	21 consecutive days.	**Canagliflozin-treated vs. control** Heart weight-to-body weight ratio (HW/BW) Serum cTnT levels, Pathological scores of cardiac sections-key indicators of myocarditis severity. Left ventricular ejection fraction (LVEF) Left ventricular fractional shortening (LVFS) Left ventricular end-systolic diameter (LVIDs)	(58)

Zhao et al. (56) investigated the renal protective effects and underlying mechanisms of SGLT2 inhibitors in LN using the MRL/lpr mouse model. The experimental cohort, comprising 10-week-old MRL/lpr mice (n=10), received oral Empagliflozin at a dosage of 10 mg/kg daily for 10 weeks, while the control group (n=10) was administered an equivalent volume of 0.5% carboxymethyl cellulose sodium (**Table 1**). Comparative analysis revealed that Empagliflozin-treated mice exhibited significantly reduced levels of anti-dsDNA IgG antibodies, serum creatinine, and proteinuria. Renal histopathological assessment demonstrated a marked reduction in glomerular and tubulointerstitial damage following Empagliflozin treatment. Transcriptome analysis indicated a downregulation of inflammatory pathways in Empagliflozin-treated MRL/lpr mice. Further cellular and animal studies revealed that Empagliflozin attenuated the expression of the NOD-like receptor pyrin domain-containing protein 3 (NLRP3), caspase-1, cleaved caspase-1, and interleukin -1β(IL-1β), mitigated podocyte damage, and enhanced autophagy through inhibition of mammalian target of rapamycin complex 1 (mTORC1) activity. Immunohistochemical analysis of renal biopsy specimens from both LN patients and MRL/lpr mice demonstrated increased SGLT2 expression co-localized with reduced synaptopodin levels, which were reversed by Empagliflozin treatment. Additionally, a retrospective study involving nine LN patients treated with SGLT2 inhibitors for over 2 months indicated a significant reduction in proteinuria ranging from 29.6% to 96.3%. Throughout the treatment period, the estimated estimated glomerular filtration rate (eGFR) remained stable. These findings reinforce the renal protective effects of SGLT2 inhibitors in MRL/lpr mice and provide further evidence supporting the potential of non-immunosuppressive therapies to enhance renal function in autoimmune nephropathies such as lupus.

To evaluate the safety of SGLT2 inhibitors in patients with SLE, a single-arm, open-label Phase I/II trial in Chinese patients with SLE, including with and without LN by Wang et al. (55) assessed the safety and efficacy of dapagliflozin (**Table 1**). The study enrolled 38 SLE patients who received dapagliflozin (10 mg/day) in addition to standard therapy for a duration of 6 months. The primary endpoint focused on safety, while secondary endpoints included efficacy assessments, specifically disease activity. Analysis of the primary endpoint revealed a total of 19 adverse events (AEs), of which 7 (18.42%) were related to disease flare-ups and 12 (31.58%) were attributed to dapagliflozin. Secondary endpoint analysis did not show significant improvements in SLE Disease Activity Index scores or proteinuria among the 17 LN patients. Notably, overall eGFR remained stable during the treatment period, and LN patients with a baseline eGFR <90 mL/min/1.73 m² demonstrated an improved eGFR slope over 6 months, suggesting a potential renal protective effect of SGLT2 inhibitors in LN patients with pre-existing renal impairment (CKD stage 2 or higher). This study indicates that dapagliflozin has an acceptable safety profile in adult SLE patients. Further investigation is warranted to elucidate its potential renal protective effects and long-term safety in SLE patients, particularly those with LN.

A multicenter cohort study leveraging the U.S. TriNetX clinical data platform investigated a cohort of 31,790 patients diagnosed with both SLE and T2DM between January 1, 2015, and December 31, 2022. Through 1:1 propensity score matching, the study identified 1,775 patients using SGLT2 inhibitors and 1,775 non-users. Kaplan-Meier methods and Cox proportional hazards regression models were employed to calculate adjusted hazard ratios (AHR) over a 5-year period for outcomes including LN, dialysis, kidney transplantation, heart failure (HF) and all-cause mortality. The results demonstrated that SGLT2 inhibitors users had significantly lower risks compared to non-users: LN (AHR 0.55; 95% CI 0.40-0.77), dialysis (AHR 0.29; 95% CI 0.17-0.48), kidney transplantation (AHR 0.14; 95% CI 0.03-0.62), HF (AHR 0.65; 95% CI 0.53-0.78) and all-cause mortality (AHR 0.35; 95% CI 0.26-0.47) (59). These findings suggest that SGLT2 inhibitors may offer substantial renal and cardiovascular protective benefits for patients with SLE and T2DM.

In parallel, Benjamin et al. (60) reviewed the literature and suggested that SGLT2 inhibitors could alleviate the chronic effects of LN on both renal and cardiovascular systems (61). The review recommended combining SGLT2 inhibitors with angiotensin-converting enzyme (ACE) inhibitors or angiotensin receptor blockers following the stabilization of kidney function through appropriate immunosuppressive therapy. Additionally, SGLT2 inhibitors show promise in addressing various SLE-related complications, including pulmonary hypertension, metabolic syndrome, and hypertension (62).

Despite the inherent limitations of the studies reviewed, there is cautious optimism that SGLT2 inhibitors, recognized for their potent cardiorenal protective effects, will confer significant therapeutic benefits to SLE patients, potentially enhancing immune regulation alongside standard immunosuppressive treatments. Anticipation is high for forthcoming larger-scale randomized controlled trials to further validate the therapeutic efficacy of SGLT2 inhibitors in SLE. Several ongoing trials registered on clinicaltrials.gov (e.g., NCT06155604, NCT06113900, NCT05748925, NCT05704088) (**Table 1**) are eagerly awaited to provide additional insights into the efficacy and safety of SGLT2 inhibitors in this patient population.

### 3.2 SGLT2 inhibitors and immunoglobulin A nephropathy

IgAN is a prevalent primary glomerular disease characterized by the deposition of IgA in the mesangium, which often progresses to ESRD despite treatment with RAASi and immunosuppressants (63). Excitingly, in a notable study, Wheeler et al. conducted a multicenter, double-blind, placebo-controlled, randomized clinical trial (Dapagliflozin-CKD, NCT03036150) to evaluate the efficacy of dapagliflozin in improving renal and cardiovascular outcomes in CKD patients with or without T2DM (64). The trial included 270 patients with IgAN, 254 of whom (94%) had diagnosis confirmed by kidney biopsy. Participants were randomized to receive either dapagliflozin (n=137) or placebo (n=133), with both groups demonstrating comparable baseline characteristics and a median follow-up duration of 2.1 years (**Table 1**). The primary composite endpoint was defined as a 50% or greater decline in eGFR (verified by a second serum creatinine measurement at least 28 days apart), progression to ESRD (characterized by maintenance dialysis for at least 28 days, kidney transplantation, or an eGFR <15 mL/min/1.73 m² confirmed by a second measurement at least 28 days apart), or death attributable to kidney or cardiovascular causes. Secondary endpoints focused on kidney-specific outcomes. Results revealed that dapagliflozin significantly reduced the primary composite endpoint, with 6 participants (4%) in the dapagliflozin group compared to 20 participants (15%) in the placebo group (HR 0.29; 95% CI 0.12–0.73; P=0.005). Similarly, secondary kidney-specific outcomes were favorable (HR 0.24; 95% CI 0.09–0.65; P=0.002). Progression to ESRD occurred in 5 participants (4%) in the dapagliflozin group versus 16 participants (12%) in the placebo group (HR 0.30; 95% CI 0.11–0.83; P=0.014). The mean annual decline in eGFR was -3.5 mL/min/1.73 m² in the dapagliflozin group versus -4.7 mL/min/1.73 m² in the placebo group. Additionally, dapagliflozin reduced the urine albumin-to-creatinine ratio by 26% relative to placebo. Importantly, dapagliflozin was well-tolerated with no new Adverse drug event (ADE) reported in this cohort. These findings suggest that dapagliflozin effectively mitigates the risk of CKD progression in IgAN patients, providing early evidence that SGLT2 inhibitors may serve as a safe and beneficial adjunct to existing standard treatments for IgAN.

Similarly, the Empagliflozin -KIDNEY (65) trial demonstrated that the addition of Empagliflozin significantly reduces the risk of composite outcomes, such as progression of kidney disease and cardiovascular death, in individuals with CKD. Of particular note, this study included a considerable cohort of patients with IgAN (n=817), thereby supporting the potential application of SGLT2 inhibitors as a promising novel therapeutic strategy for managing IgAN (41, 66) (**Table 1**).

### 3.3 SGLT2 inhibitor and idiopathic membranous nephropathy

IMN is a prevalent AIDs characterized by organ-specific pathology and a leading cause of nephrotic syndrome in adults, with a significant progression to ESRD (67). Evidence underscores that immune complex deposition is central to the pathogenesis of IMN, with Th17/Treg cell dysregulation and Th1/Th2 polarization imbalance being closely linked to disease onset (68, 69). According to reports, SGLT2 inhibitors have immunomodulatory effects and can improve the Th17/Treg cell imbalance in diabetic mice (70).

Given the renal protective and immunomodulatory properties of SGLT2 inhibitors, Lv et al. investigated the effects of Canagliflozin on urinary protein levels and renal histopathology in a rat model of membranous nephropathy (MN) (57). In this study, Sprague-Dawley (SD) rats were allocated to four groups: normal control, MN model, Canagliflozin -treated (10 mg/kg/day), and losartan-treated (10 mg/kg/day), with six rats per group. All rats received oral treatment for 8 weeks. Canagliflozin administration resulted in significant reductions in urine total protein/creatinine ratios by 56.3% (P < 0.01) and 69.8% (P < 0.01) at 4 and 8 weeks, respectively. Serum albumin levels increased by 26.3% (P < 0.05) and 31.8% (P < 0.05), respectively (**Table 1**). Additionally, Canagliflozin ameliorated glomerular pathological damage, reduced renal immune complex deposition, and mitigated podocyte injury more effectively than losartan. To explore the potential immunomodulatory mechanisms of Canagliflozin, the study examined changes in peripheral blood T lymphocyte subsets. Results indicated that Canagliflozin treatment enhanced the proportion of Th1 cells by 2.3-fold, reduced Th2 cells by 68.5%, and significantly inhibited IgG1 secretion in B cells as well as immune complex deposition beneath the glomerular epithelium. Co-culture experiments revealed that B cells from MN rats activated mTOR and ULK1 phosphorylation in podocytes, leading to impaired podocyte autophagy and injury. Canagliflozin treatment reversed these pathological changes in co-cultured B cells, suggesting that Canagliflozin’s renal protective effects stem from correcting Th1/Th2 cell imbalances and restoring autophagy in podocytes inhibited by abnormal IgG secretion.

Furthermore, Hammad et al. conducted a randomized controlled trial to evaluate the potential benefits of SGLT2 inhibitors in patients with immune-mediated kidney diseases (71). The study enrolled 50 patients with nephropathies including SLE (24%), minimal change disease (20%), MN (16%), and focal segmental glomerulosclerosis (12%). Participants were randomized 1:1 to receive either Empagliflozin (25 mg/day) or placebo, alongside RAAS inhibitors and immunosuppressants. The primary endpoints were changes in creatinine levels, eGFR, and proteinuria after three months. While Empagliflozin did not significantly improve eGFR, it notably reduced proteinuria levels, indicating its potential therapeutic benefit in managing immune-mediated kidney diseases.

### 3.4 SGLT2 inhibitor and experimental autoimmune myocarditis

Myocarditis is a severe inflammatory condition of the heart, a leading cause of sudden cardiac death among children and young adults globally (72, 73). Autoimmune-mediated cardiac injury is increasingly recognized as a pivotal factor in the pathogenesis of myocarditis (74) The experimental autoimmune myocarditis (EAM) model is widely utilized to elucidate the immunological mechanisms underlying myocardial damage.

In a study by Qi Long et al (58) (**Table 1**). the efficacy of Canagliflozin was assessed in a MyHC-α peptide-induced EAM mouse model. Mice received Canagliflozin (30 mg/kg/day) or saline for 21 consecutive days. The EAM group exhibited significant elevations in the heart weight-to-body weight ratio (HW/BW), serum cTnT levels, and pathological scores of cardiac sections-key indicators of myocarditis severity (all P < 0.05). Notably, these indices of cardiac damage were significantly ameliorated in the CANA -treated group (all P < 0.05). Echocardiographic analysis revealed that, compared to the EAM group, the Canagliflozin group demonstrated enhanced left ventricular ejection fraction (LVEF) and left ventricular fractional shortening (LVFS), alongside a reduction in left ventricular end-systolic diameter (LVIDs) (all P < 0.01). Moreover, Canagliflozin treatment led to a marked downregulation of NLRP3 inflammasome complex components (including NLRP3, ASC, and Caspase-1) and their downstream mediators (IL-1β and IL-18), as well as a reduction in Th17 cell infiltration within the heart. Canagliflozin also significantly decreased the Bax/Bcl-2 ratio, cleaved Caspase-3 protein levels, and the percentage of TUNEL-positive myocardial cells—markers indicative of apoptosis. These findings suggest that Canagliflozin may offer significant therapeutic potential for the management of myocarditis by modulating immune responses and mitigating myocardial damage.

## 4 The potential mechanism of SGLT 2 inhibitors in AIDs

### 4.1 Inhibition of T cell proliferation and activation

T lymphocytes are pivotal in adaptive immune responses, recognizing antigens through surface antigen receptors and initiating a cascade of signal transduction and metabolic changes to support their proliferation, differentiation, and effector functions (75). Dysregulated T cell activation is a hallmark of AIDs, often driven by the heightened metabolic demands of these cells (76). Consequently, targeting T cell metabolism represents a promising therapeutic approach in autoimmune conditions. Recent research has highlighted that SGLT2 inhibitors exert notable off-target effects, such as inhibiting mitochondrial glutamate dehydrogenase (GDH) and complex I (77, 78). Beyond these effects, SGLT2 inhibitors like dapagliflozin also exhibit immunomodulatory properties, evidenced by their ability to correct the Th17/Treg cell imbalance in diabetic mice (70). A recent study further elucidates the immunomodulatory potential of SGLT2 inhibitors, specifically Canagliflozin. In this study, Jenkins et al. (60) investigated the impact of Canagliflozin on human CD4^+^ naive T cells, activated with anti-CD3 and anti-CD28 in the presence of physiologically relevant doses of Canagliflozin. Their findings revealed that Canagliflozin significantly reduced IL-2 production in a dose-dependent manner and curtailed T cell activation by decreasing the expression of activation markers such as CD25, CD44, and CD69.

**Figure 3:**
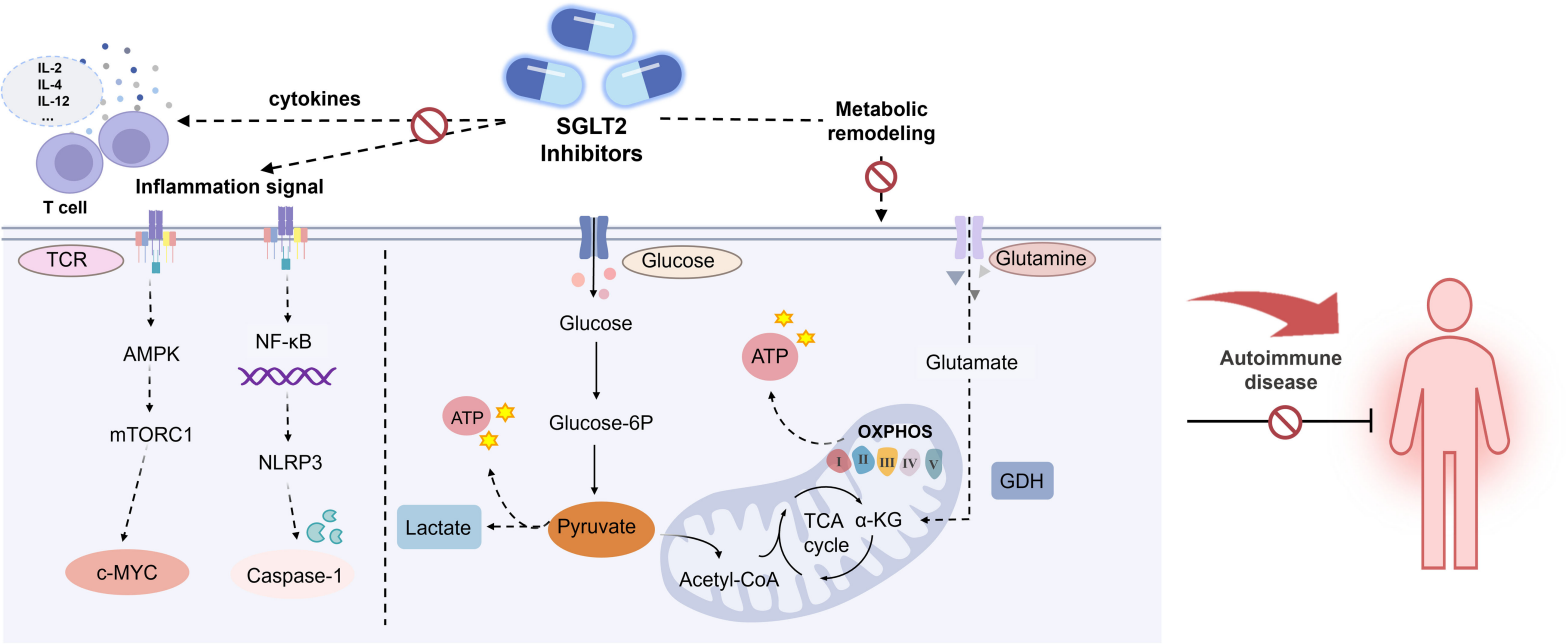
Effects of SGLT2 inhibitor in AIDs. AMPK, AMP-activated protein kinase; MTORC1, Mechanistic target of rapamycin complex 1. c-MYC, Cellular-myelocytomatosis viral oncogene; NLRP3, NOD-like receptor thermal protein domain associated protein 3; NF-κB, Nuclear factor kappa-B; GDH, Glutamate dehydrogenase.

Moreover, Canagliflozin was shown to impair T cell proliferation, as evidenced by the downregulation of mTORC1 activity (60) (**Figure 3**). AMP-activated protein kinase (AMPK), a key regulator of bioenergetic metabolism, influences immune cell function through its downstream pathways. In the context of autoimmunity, T cells undergo metabolic reprogramming to meet the increased energy demands for activation and differentiation, with AMPK playing a central role (79). For instance, mTOR, a downstream target of AMPK, is a crucial regulator of Treg and Th17 cell differentiation. It orchestrates glycolysis by modulating key transcription factors, and its activity impacts cellular immune responses and metabolic demands. As a major driver of glycolysis, mTORC1 enhances glycolytic processes by upregulating glucose transporter 1 (GLUT1) and suppressing Treg upregulation.

In this study, markers of mTORC1 activity, including ribosomal protein S6 (RPS6) and eukaryotic translation initiation factor 4E-binding protein 1 (4E-BP1) phosphorylation, were reduced at 4 hours and 24 hours post-activation. In contrast, AMPK activity and its downstream target acetyl-CoA carboxylase remained unchanged, indicating that Canagliflozin alters T cell signaling by diminishing early downstream receptor target phosphorylation, which in turn significantly impairs T cell proliferation. These findings are corroborated by studies in autoimmune patient cohorts, such as those with SLE and RA, which confirm Canagliflozin ‘s T cell suppressive effects (60).

### 4.2 Inhibition of T cell metabolic reprogramming

Upon activation of the T cell receptor (TCR), a notable metabolic shift occurs, characterized by enhanced glycolysis and the upregulation of glucose transporter proteins, such as GLUT1 (80), to facilitate increased glucose uptake. Concurrently, mitochondrial metabolism in T cells undergoes significant alterations, impacting OXPHOS and increasing mitochondrial mass. Metabolic reprogramming influences not just the activation and proliferation of T cells, but also the differentiation pathways of T cell subsets, which have been shown to play major regulatory roles in multiple autoimmune disorders. Therefore, metabolic reprogramming is emerging as a new therapeutic target for aid.

Recent studies have highlighted the impact of SGLT2 inhibitors on these metabolic processes (**Figure 3**). For instance, Empagliflozin has been shown to inhibit mTORC1 signaling by binding and obstructing GLUT1 and upregulating glucose transporter 4 (GLUT4) in myocardial cells, leading to a reduction in intracellular glucose levels (81). Similarly, Canagliflozin decreases ATP production derived from glycolysis and significantly reduces ATP generation through OXPHOS. Canagliflozin also impairs TCA cycle metabolism by inhibiting mitochondrial GDH, further contributing to altered cellular metabolism (60).

Previous studies have revealed that the transcription factor c-Myc plays a pivotal role in regulating glucose metabolism by upregulating key enzymes and molecules involved in glycolysis, including GLUT1, hexokinase, phosphofructokinase, and glutaminase. Given its central role, the absence of c-Myc impairs the activation and function of CD4^+^ and CD8^+^ T cells. Research indicates that treatment with Canagliflozin attenuates c-Myc signaling, leading to mitochondrial dysfunction and disrupting T cell metabolism and functionality. Furthermore, Canagliflozin treatment significantly inhibits various c-Myc-related metabolic targets, such as GLUT1, hexokinase 2, dihydrofolate reductase, ATP citrate lyase, and fatty acid synthase, underscoring its role in modulating T cell metabolism through the c-Myc pathway (60).

### 4.3 Improvement of chronic inflammatory response

AIDs are characterized by systemic or organ-specific inflammation, wherein immune cells play a pivotal role in establishing a pro-inflammatory autoimmune milieu by recognizing and responding to self-antigens presented by antigen-presenting cells (82). Upon TCR activation, a series of signaling cascades lead to the upregulation of activation markers, culminating in the expansion of Teff that produce critical pro-inflammatory cytokines and other inflammatory mediators (83). Cytokines such as interleukin-12 (IL-12), IFN-γ, IL-4, and Transforming growth factor-beta (TGF-β) directly influence the differentiation of CD4^+^ T cells into various effector T helper cell subsets, which is essential for maintaining immune tolerance and function (84). In a study involving CD4^+^ T cells isolated from patients with SLE and RA, activation in the presence or absence of Canagliflozin demonstrated that Canagliflozin effectively inhibited the production of IL-2, IFN-γ, interleukin-17 (IL-17), and TNF cytokines (85).

NLRP3 inflammasome, expressed in granulocytes, macrophages, T lymphocytes, and B lymphocytes, plays a crucial role in autoimmune pathogenesis. Upon activation, it triggers inflammatory cascades and cytokine release, contributing to tissue damage and the development of various AIDs (86, 87). In RA patients, NLRP3 inflammasome activation in CD4^+^ T cells promotes Th17 cell differentiation via IL-1β production, underscoring its significance in RA pathology. Clinical trials have reported elevated expression levels of NLRP3-related proteins in the blood of RA patients, with further upregulation following NLRP3 activation (88, 89). In LN patients, NLRP3 inflammasome activation has been linked to podocyte injury and proteinuria. Research indicates that SGLT2 inhibitors can attenuate lipopolysaccharide-induced and NLRP3-mediated inflammatory responses, modulate macrophage polarization via interactions with mTOR and AMPK pathways (90) (**Figure 3**). Empagliflozin, in particular, reduces the activity of NLRP3, caspase-1, cleaved caspase-1, and IL-1β in MRL/lpr mice, thereby mitigating podocyte injury. Furthermore, Empagliflozin enhances autophagy by reducing mTORC1 signaling, which further alleviates podocyte damage in LN mice (56). Thus, SGLT2 inhibitors hold potential for mitigating inflammation, regulating endothelial dysfunction, and attenuating atherosclerosis, which are relevant to the pathophysiology of SLE.

In the EAM mouse model (58), Canagliflozin significantly ameliorates cardiac inflammation induced by myocarditis through inhibition of NLRP3 inflammasome activation, reduction of IL-17 secretion, and modulation of CD4^+^ T cell differentiation, particularly Th17 cells. The authors propose that SGLT1 in the myocardium may contribute to Canagliflozin ‘s beneficial effects in EAM, potentially by inhibiting SGLT1 and Na^+^/Ca2^+^ exchanger 1 (NCX1) signals. This action may improve lysosomal function, promote autophagosome degradation, and inhibit NLRP3 inflammasome assembly. However, the precise regulatory mechanisms of Canagliflozin in EAM warrant further investigation to validate these findings.

## 5 Conclusions

AIDs represent a category of immune disorders wherein the immune system erroneously targets and damages its own tissues and organs. Emerging therapeutic strategies increasingly focus on modulating T cell immune metabolism as a novel approach to managing these conditions. SGLT2 inhibitors, a recent class of oral hypoglycemic agents, not only effectively lower blood glucose levels but also exert significant effects on T cell function by altering T cell metabolism. This mechanism presents promising therapeutic potential for treating AIDs. Moreover, the favorable safety profile and substantial cardio-renal protective benefits of SGLT2 inhibitors suggest a promising outlook for their application in AIDs. Current clinical research predominantly investigates the efficacy of SGLT2 inhibitors in conditions such as LN, IMN, and IgAN. However, there is a notable paucity of studies exploring the efficacy of SGLT2 inhibitors in other autoimmune disorders, such as RA, MS and Sjögren’s syndrome, future clinical studies are warranted in these areas.

It is crucial to recognize that while Empagliflozin, Canagliflozin, Dapagliflozin, and Ertugliflozin are all classified as SGLT2 inhibitors, their structural differences result in varied physiological and pharmacological properties. For instance, Empagliflozin, dapagliflozin, and ertugliflozin are highly selective for SGLT2. Conversely, sotagliflozin and luseogliflozin are dual inhibitors of both SGLT-1 and SGLT2, exhibiting potent inhibition of both transporters. Canagliflozin, however, demonstrates weaker selectivity for SGLT2 compared to other SGLT2 inhibitors and has additional off-target effects, including direct mitochondrial actions, which may influence its efficacy in glucose reduction, cardio-renal protection, and immunomodulation. Notably, although SGLT2 inhibitors show great potential in AIDs, the clinical evidence regarding the effectiveness of this class of drugs in AIDs is still limited. Most of the studies rely on small sample sizes and short-term follow-ups, particularly in autoimmune nephropathies. Thus, more long-term and large-scale studies are needed to confirm their clinical benefit.
